# Treatment of childhood phthiriasis palpebrarum with systemic ivermectin

**DOI:** 10.1016/j.jdcr.2021.05.034

**Published:** 2021-06-03

**Authors:** Ibrahim Fouda, Ahmad Nofal, Mohamed M. Fawzy, Fatma Eldeeb

**Affiliations:** a Dermatology, Venereology and Andrology Department, Damietta Faculty of Medicine, Al-Azhar University, Cairo, Egypt; b Member of Interactive Dermatology Research Group, Cairo, Egypt; c Dermatology, Venereology and Andrology Department, Faculty of Medicine, Zagazig University, Zagazig, Egypt; d Dermatology, Venereology and Andrology Department, Faculty of Medicine, Tanta University, Tanta, Egypt

**Keywords:** crab louse, ivermectin, phthiriasis palpebrarum

## Introduction

Phthiriasis palpebrarum is an ectoparasitosis of the eyelashes due to infestation with *Phthirus pubis*, also known as crab louse. It may affect children or adults and present with different clinical symptoms such as eyelid itching, pain, or gritty sensation. Adult lice and nits can be detected by a careful clinical examination and confirmed by dermoscopy. The treatment of phthiriasis palpebrarum may be in the form of topical application of ovidicidal formulations or physical agents, which may be combined with systemic ivermectin.^1^ Herein, we present a rare case of unilateral phthiriasis palpebrarum affecting an 8-year-old child with an excellent response to oral ivermectin.

## Case report

**Fig 1:**
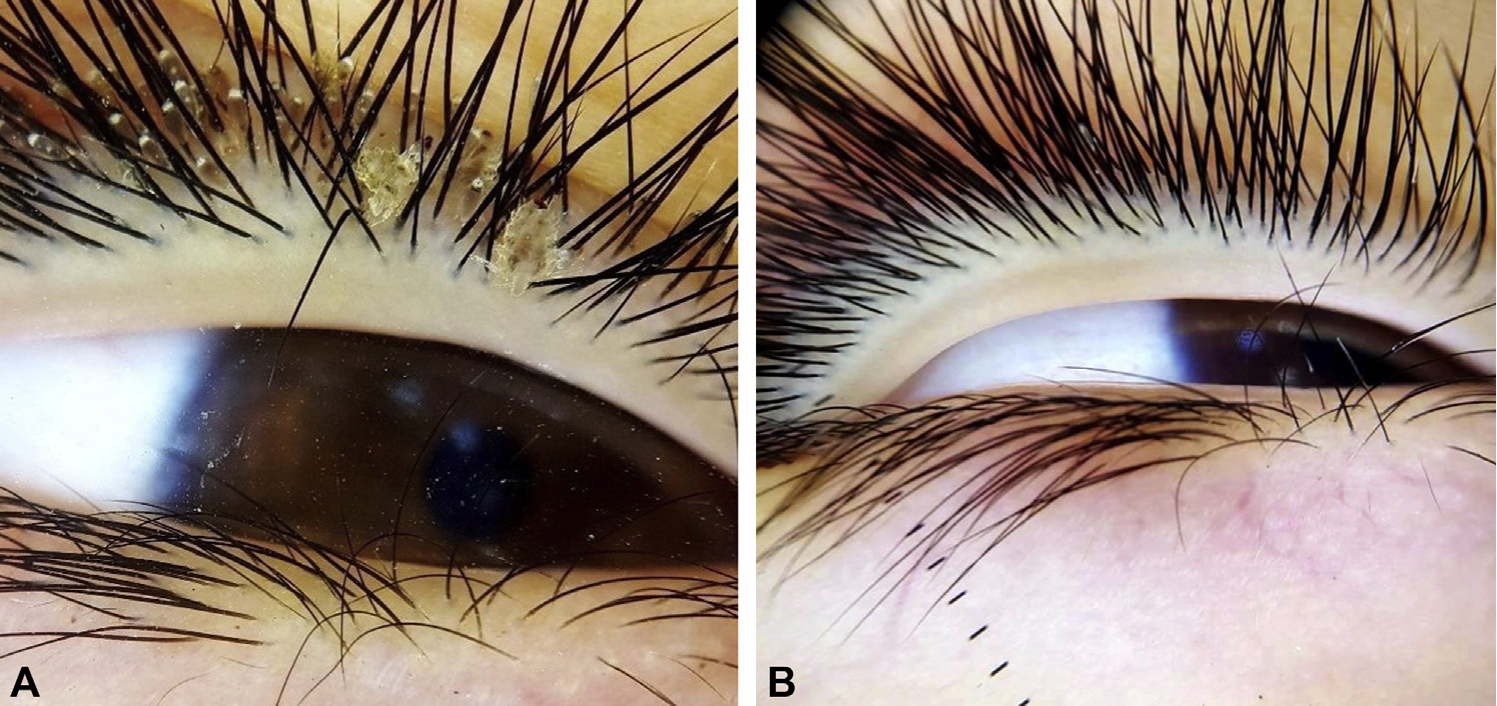
An 8-year-old boy with multiple nits and crab lice attached to the right eye lashes. **A,** Dermoscopy showing the detailed features of the lice and its nits attached to the side of eyelashes. **B,** Complete cure after 2 successive doses of systemic ivermectin and topical petroleum jelly application as shown by dermoscopic examination.

**Fig 2:**
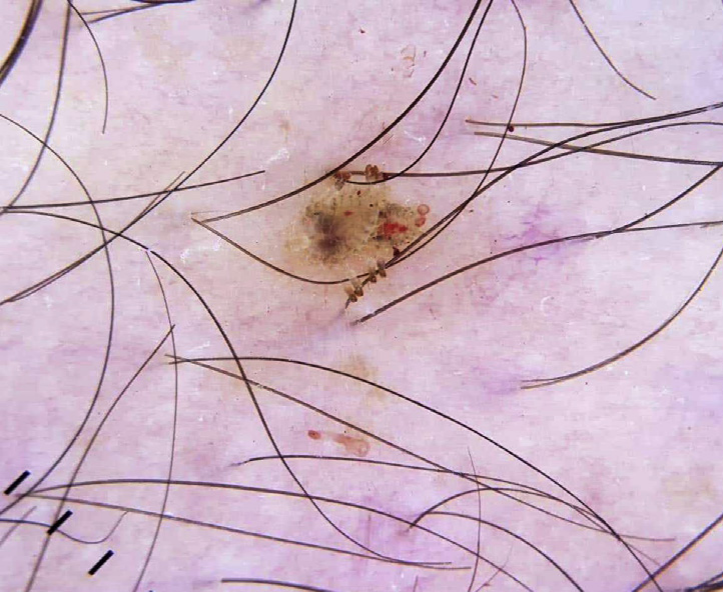
Pediculosis infestation of the grandfather: dermoscopic examination showing the characteristic crab louse.

An 8-year-old boy presented to our dermatology outpatient clinic with symptoms of itching, irritation, and redness on his right eyelid for >2 months. Clinical and dermoscopic examination revealed the presence of multiple nits attached to the eye lashes, and more than 3 crab lice were also found (Fig 1, *A* and *B*). Family history revealed that his grandfather was treated in the same clinic with oral ivermectin for a severe infestation of pediculosis pubis 3 months prior to the presentation of his grandson (Fig 2). Based on the positive family history and the clinical and dermoscopic detection of the adult lice and nits, the diagnosis of phthiriasis palpebrarum was established. The patient was treated with oral ivermectin 200 μg/kg/week for 2 successive weeks, resulting in almost an complete eradication of the infestation, leaving behind a few nits that were cleared by the topical application of petrolatum jelly (Fig 1, *B*). No recurrence was detected after a 6-month follow-up period.

## Discussion

Lice are obligate parasites that feed solely on the blood of the infested hosts. Human lice include body louse (*Pediculus humanus corporis*), head louse (*Pediculus humanus capitis*), and pubic louse (*Phthirus pubis*). The pubic louse gets the nickname of “crab” from its short, broad body (0.8-1.2 mm) and large front claws, which give it a crab-like appearance that makes it possible for the pubic lice to grasp the widely spaced pubic hair. Although the pubic louse mostly infest the pubic hair, it can also spread to involve other hair-bearing areas, including eyebrows and eyelashes.^1^

Phthiriasis palpebrarum, sometimes referred to as phthiriasis ciliaris, is an uncommon cause of blepharoconjunctivitis, in which *Phthirus pubis* infests the eyelashes. Phthiriasis palpebrarum is predominantly diagnosed in children as was the case in our patient; however, its prevalence is uncertain.^2^

The most common primary symptom of phthiriasis palpebrarum is eye lid pruritus, which may be explained by the cutaneous hypersensitivity to the saliva of the louse. Other less frequently encountered symptoms are pain, burning sensation, gritty sensation, and crusty excretions from the eyelashes. Phthiriasis palpebrarum may be confused with other forms of blepharitis, especially, seborrheic blepharitis.^3^

*Phthirus pubis* infestation occurs primarily through intercourse. Therefore, once the diagnosis of phthiriasis palpebrarum in children is confirmed, detection of the source of infection is mandatory to rule out sexual abuse. The infestation may also take place through indirect routes such as sharing sleep arrangements with infested people or contact with fomites.^2^^,^^3^

In our case, the grandfather was the source of infestation with *Phthirus pubis* that resulted in a severe infestation involving all the hair-bearing areas. Unfortunately, the patient was misdiagnosed, leading to the transmission of the infection to his grandson. The grandfather was successfully treated at our clinic with systemic ivermectin at a dose of 200 μg/kg, once weekly for 2 successive weeks, to confirm the eradication of the severe infestation.

Due to the absence of terminal hair in other body regions in children, as was the case in our patient, eyelashes may be one of the most common areas of localization of *Phthirus pubis* in children, thus causing blepharoconjunctivitis.^4^ Phthiriasis palpebrarum in adults has been commonly reported; however, unilateral eyelash involvement, particularly in children, is rarely reported in the literature.^5^

The identification and diagnosis of crab lice is by dermoscopy, which is a simple and noninvasive tool that can also aid in the monitoring of the treatment response by assessing whether the nits are empty or still contain viable or dead nymphs.^1^

Many therapeutic options are available for the treatment of phthiriasis palpebrarum, including manual removal of the adult lice and nits using forceps, trimming of eyelashes, argon laser therapy, 1% gamma-benzene hexachloride cream, 1% mercuric oxide ointment, physostigmine eye ointment, petrolatum ointment, pilocarpine 4% gel, malathion shampoo or drops 1%, permethrin 5% ointment, and 20% fluorescein solution.^6^

Petrolatum jelly is not ovicidal and its mechanism of action is still unclear. However, it covers the lice and blocks the breathing holes, thereby preventing their breathing or movement.^7^ In this case, petrolatum jelly was used to remove the remaining nits and not to kill the adult lice.

Ivermectin is an anthelmintic agent that has proven to be effective for the treatment of onchocerciasis, loiasis, strongyloidiasis, scabies, and pediculosis, particularly head lice.^8^ Although the successful use of ivermectin for the treatment of phthiriasis palpebrarum was not evident in the dermatologic literature, it has been found in 2 ophthalmologic reports.^9^^,^^10^ Ivermectin acts by blocking the chemical transmission across nerve synapses that use glutamate or gamma-aminobutyric acid, which are the neurotransmitters for peripheral motor function in lice, resulting in paralysis and death of the parasite.^9^

Because ivermectin is not ovicidal and its plasma half-life is 16 hours, a single dose may be inadequate to eradicate the different stages of the parasite and a second dose is usually recommended after 1 week to kill the newly hatched nymphs. One of the potential advantages of systemic ivermectin is its high compliance compare to the topical formulations that commonly cause irritation at the site. On the other hand, oral ivermectin is contraindicated in children <5 years old and/or weighing <15 kilograms to avoid the potential ability of the drug to cross the poorly developed blood-brain barrier at this age.^8^^,^^9^

In conclusion, we present a rare case of unilateral pediatric phthiriasis palpebrarum that showed an excellent response to 2 doses of oral ivermectin that seems to be a promising therapeutic option for this challenging lice infestation.

## Conflicts of interest

None disclosed.
